# Considerations for higher efficiency and productivity in research activities

**DOI:** 10.1186/s13040-016-0115-3

**Published:** 2016-11-09

**Authors:** Diego A. Forero, Jason H. Moore

**Affiliations:** 1 Laboratory of NeuroPsychiatric Genetics, Biomedical Sciences Research Group, School of Medicine, Universidad Antonio Nariño, Bogotá, Colombia; 2 PhD Program in Health Sciences, School of Medicine, Universidad Antonio Nariño, Bogotá, Colombia; 3 Department of Biostatistics and Epidemiology, Institute for Biomedical Informatics, Perelman School or Medicine, University of Pennsylvania, Philadelphia, PA 19104-6116 USA

**Keywords:** Scientometrics, Biomedical research, Bibliometrics, Global science, Scientific productivity, Academic mentoring

## Abstract

There are several factors that are known to affect research productivity; some of them imply the need for large financial investments and others are related to work styles. There are some articles that provide suggestions for early career scientists (PhD students and postdocs) but few publications are oriented to professors about scientific leadership. As academic mentoring might be useful at all levels of experience, in this note we suggest several key considerations for higher efficiency and productivity in academic and research activities. More research is needed into the main work style features that differentiate highly productive scientists and research groups, as some of them could be innate and others could be transferable. As funding agencies, universities and research centers invest large amounts of money in order to have a better scientific productivity, a deeper understanding of these factors will be of high academic and societal impact.

As research funding becomes less abundant and more competitive, it is more important than ever to focus on efficiency and productivity. This is because funding agencies want to see their limited resources have a bigger impact and researchers need increased productivity to compete for highly-prized research grants. There are several factors that are known to affect research productivity [1, 2]; some of them imply the need for large financial investments and others are related to work styles [3, 4]. Significant economic resources are dedicated to teaching technical research abilities in PhD programs, but few courses are oriented to the consideration of the key elements needed for scientific productivity. As academic mentoring might be useful at all levels of experience [5, 6], in this note we suggest several key considerations for higher efficiency and productivity in academic and research activities.

1. **Unnecessary meetings disrupt scientific productivity.** Usually, the daily agenda of a University professor is plagued of long meetings. In many cases, those face-to-face meetings might be replaced by more efficient strategies, such as the use of online tools for collaborative writing or for polling of opinions. In other cases, the use of a predefined and short agenda could lead to shorter and more productive meetings and provide more time for scientific activities [7].
2. **Lack of clear priorities affects science.** As in other highly competitive areas, having clear and consistent priorities is needed. The adherence to those priorities should be evident in daily activities, avoiding to dedicate too much time (more than needed or planned) on other tasks (for example, teaching or administrative duties) [8] and being able to work hard and constantly on research topics.
3. **Having more than one job is good for money but negative for productivity.** In certain areas it is common that professionals have more than one job, particularly due to economic reasons. Although it could lead to a higher income, it is usually negative for scientific productivity and quality of life. A scientific career implicates the need for a dedication that is going beyond 40 h per week, to be able to work hard and focus on research activities [9].
4. **Depending too much on collaborations is undesirable.** A fundamental aspect of becoming an independent scientist is the possibility of leading projects and initiatives, which implies the transition to becoming a principal investigator in research projects and a corresponding author in articles. Although scientists that depend highly on publications led by others might appear as productive independent researchers (high publication count, cumulative impact factor and h-index, among others), it is easy to detect them through bibliometric analysis, a process usually carried out by funding and promotion committees [10, 11].
5. **Lack of collaborations is negative for research.** Science is currently characterized by interdisciplinary, interinstitutional and international collaborations [12]. Scientists that are reluctant to collaborate with other groups have a lower probability of getting large grants or being published in international journals. Some personal myths and fears that make difficult the development of collaborations might be overcome [13, 14].
6. **Aiming too high or too low is counterproductive.** Research projects that do not aim to incorporate innovative processes or to study novel topics have more issues in getting funded or published. On the other hand, of particular relevance for scientists in developing countries, the strategic planning of a research lab needs to be adjusted to the reality of the context, taking into account the available local resources [15, 16]. In many cases, the scientific endeavor is a gradual process, starting with small projects and moving to larger proposals.
7. **Never become tired of writing grants or papers.** Usually, funds from competitive grants are the main fuel for a laboratory. If a professor becomes tired of writing grant applications it would mean the end of his funding [17]. The same applies to writing papers [18]. Resilience against internal failures or bad external reviews is an important trait in science and future success in grant applications will depend on the products generated by your current research projects (excuses or complaints are not scientific products).
8. **Do not delegate being the Principal Investigator.** An adequate and constant supervision of lab members is fundamental for a satisfactory functioning of a research group. It is possible to delegate some administrative, scientific and academic duties, but the general supervision and direct leadership of a laboratory is of paramount importance, taking into account the scientific, ethical and administrative implications of delegating key responsibilities to personnel without the adequate training or experience.
9. **Try to create a positive working environment.** Selection of the best scientific personnel available is key for scientific success and an adequate nurturing of lab members usually creates a positive working environment [7]. Young scientists that enjoy research and that are open to receive adequate training and supervision are a key asset in a group.

There are some articles that provide suggestions for early career scientists (PhD students and postdocs) [19, 20] but few publications are oriented to professors [21] about scientific leadership. More research is needed into the main work style features that differentiate highly productive scientists and research groups, as some of them could be innate and others could be transferable [3, 22–25]. As funding agencies, universities and research centers invest large amounts of money in order to have a better scientific productivity, a deeper understanding of these factors will be of high academic and societal impact [4, 26–28].

## References

[1] Moses H, Matheson DH, Cairns-Smith S, George BP, Palisch C, Dorsey ER (2015). The anatomy of medical research: US and international comparisons. JAMA.

[2] Gantman ER (2012). Economic, linguistic, and political factors in the scientific productivity of countries. Scientometrics.

[3] Wagner CS, Horlings E, Whetsell TA, Mattsson P, Nordqvist K (2015). Do Nobel Laureates Create Prize-Winning Networks? An Analysis of Collaborative Research in Physiology or Medicine. PLoS ONE.

[4] Tatsioni A, Vavva E, Ioannidis JP (2010). Sources of funding for Nobel Prize-winning work: public or private?. FASEB J.

[5] Detsky AS, Baerlocher MO (2007). Academic mentoring--how to give it and how to get it. JAMA.

[6] Sambunjak D, Straus SE, Marusic A (2006). Mentoring in academic medicine: a systematic review. JAMA.

[7] Simone JV (1999). Understanding academic medical centers: Simone’s Maxims. Clin Cancer Res.

[8] Vicens Q, Bourne PE (2009). Ten simple rules to combine teaching and research. PLoS Comput Biol.

[9] Bland CJ, Center BA, Finstad DA, Risbey KR, Staples J (2006). The impact of appointment type on the productivity and commitment of full-time faculty in research and doctoral institutions. J High Educ.

[10] Aziz NA, Rozing MP (2013). Profit (p)-index: the degree to which authors profit from co-authors. PLoS ONE.

[11] Ioannidis JP, Klavans R, Boyack KW (2016). Multiple Citation Indicators and Their Composite across Scientific Disciplines. PLoS Biol.

[12] Wagner CS, Park HW, Leydesdorff L (2015). The Continuing Growth of Global Cooperation Networks in Research: A Conundrum for National Governments. PLoS ONE.

[13] Vicens Q, Bourne PE (2007). Ten simple rules for a successful collaboration. PLoS Comput Biol.

[14] Knapp B, Bardenet R, Bernabeu MO, Bordas R, Bruna M, Calderhead B, Cooper J, Fletcher AG, Groen D, Kuijper B (2015). Ten simple rules for a successful cross-disciplinary collaboration. PLoS Comput Biol.

[15] Moreno E, Gutierrez JM (2008). Ten simple rules for aspiring scientists in a low-income country. PLoS Comput Biol.

[16] Garcia PJ, Curioso WH (2008). Strategies for aspiring biomedical researchers in resource-limited environments. PLoS Negl Trop Dis.

[17] Bourne PE, Chalupa LM (2006). Ten simple rules for getting grants. PLoS Comput Biol.

[18] Bourne PE (2005). Ten simple rules for getting published. PLoS Comput Biol.

[19] Bourne PE, Friedberg I (2006). Ten simple rules for selecting a postdoctoral position. PLoS Comput Biol.

[20] Gu J, Bourne PE (2007). Ten simple rules for graduate students. PLoS Comput Biol.

[21] Bourne PE, Barbour V (2011). Ten simple rules for building and maintaining a scientific reputation. PLoS Comput Biol.

[22] Mazloumian A (2012). Predicting scholars’ scientific impact. PLoS ONE.

[23] Boyack KW, Klavans R, Sorensen AA, Ioannidis JP (2013). A list of highly influential biomedical researchers, 1996–2011. Eur J Clin Invest.

[24] Shneider AM (2009). Four stages of a scientific discipline; four types of scientist. Trends Biochem Sci.

[25] Prpić K (2000). The publication productivity of young scientists: An empirical study. Scientometrics.

[26] Ioannidis JP, Patsopoulos NA, Kavvoura FK, Tatsioni A, Evangelou E, Kouri I, Contopoulos-Ioannidis DG, Liberopoulos G (2007). International ranking systems for universities and institutions: a critical appraisal. BMC Med.

[27] Kozak M, Bornmann L (2012). A new family of cumulative indexes for measuring scientific performance. PLoS ONE.

[28] Lowenstein SR, Fernandez G, Crane LA (2007). Medical school faculty discontent: prevalence and predictors of intent to leave academic careers. BMC Med Educ.

